# The Efficacy of Anterior Cervical Corpectomy and Fusion and Posterior Total Laminectomy on Cervical Spinal Cord Injury and Quality of Life

**DOI:** 10.1155/2022/8216339

**Published:** 2022-09-29

**Authors:** Yanlin Yin, Xinming Yang, Ye Tian, Ying Zhang, Peinan Zhang, Yongli Jia, Yao Yao, Xiuyu Du, Tianmin Li, Xiaodong Li

**Affiliations:** ^1^Department of Orthopedics, The First Affiliated Hospital of Hebei North University, Qiaoxi District, Zhangjiakou, Hebei, China; ^2^Department of Trauma, The First Affiliated Hospital of Hebei North University, Qiaoxi District, Zhangjiakou, Hebei, China; ^3^Department of General Medicine, The First Affiliated Hospital of Hebei North University, Qiaoxi District, Zhangjiakou, Hebei, China; ^4^Department of Imaging, The First Affiliated Hospital of Hebei North University, Qiaoxi District, Zhangjiakou, Hebei, China

## Abstract

This study is aimed at investigating the efficacy of anterior cervical corpectomy and fusion and posterior total laminectomy in the treatment of cervical spinal cord injury and assessing the impact of the two approaches on cervical spine function and patient quality of life. Retrospectively analyze the clinical data from 180 patients with cervical spinal cord injury who were admitted to the First Affiliated Hospital of Hebei North University from June 2019 to June 2021. The patients were divided into an anterior approach group (*n* = 89, treated with anterior cervical corpectomy and fusion) and a posterior approach group (*n* = 91, treated with posterior total laminectomy). The amount of blood loss in the posterior approach group was larger compared to the anterior approach group. Patients in the posterior approach group had higher wound diameters and operation times compared to the anterior approach group, as well as the operation cost. The visual analogue scale (VAS) scores of patients in the posterior approach group were significantly higher than in the anterior approach group one month after operation. The Japanese Orthopaedic Association (JOA), neck disability index (NDI), and American Spinal Injury Association (ASIA) scores of patients in both groups at 1, 6, and 9 months after surgery were higher compared to those before surgery, yet no significant differences were observed between the two groups. Also, no significant difference was observed in the incidence of complication and the quality of life between the two groups before and after treatment. Anterior cervical corpectomy and fusion and posterior total laminectomy can effectively restore the cervical nerve function in the treatment of cervical spinal cord injury. However, anterior subtotal vertebral resection is associated with improved perioperative indicators compared to posterior total laminectomy. Clinically, surgical methods can be selected according to imaging findings, the general condition of patients, and individual economic status.

## 1. Introduction

Cervical spinal cord injury is a common clinical indication and one of the spinal cord injuries [1]. Spinal cord injury has major impacts on the structure and function of the spinal cord that can be caused by various factors and results in autonomic, motor, and sensory nerve dysfunction. Severe cervical spine injury may result in lifelong paralysis or even death [2–4]. Recently, the incidence of clinical traumatic cervical spinal cord injury has continued to increase due to the frequent occurrence of road traffic accidents, high altitude falls and other serious accidents [5].

In cases of cervical spinal cord injury, the primary treatment goal is to reverse the nerve injury, avoid secondary injury, and restore the integrity of the spine [6]. Dehydration, nerve nutrition, prevention of nerve edema, and traction are generally adopted in the early stage of treatment of cervical spinal cord injury [7, 8]. Also, glucocorticoids are used to prevent further aggravation of cervical spinal cord injury [9].

Surgery has an important role in the management of patients with cervical spinal cord injury that is based on the imaging data and physical condition of individual patients. Due to the various mechanisms of cervical spinal cord injury, a range of surgical methods can be used. Anterior [10] or posterior approach decompression resection [11] is a common surgical method for the treatment of cervical spondylosis and has also been gradually applied in the treatment of cervical spinal cord injury. Decompression surgery can release pressure on the spinal cord with edema after injury, and the timing of surgery is particularly important to optimize recovery. The incorrect timing of surgery can lead to a slower recovery of function and even failure of the recovery of spinal cord function [12].

Anterior cervical corpectomy and fusion is a commonly used anterior approach, whilst posterior total laminectomy is a commonly used posterior surgical approach [13, 14]. These surgical methods have respective advantages and disadvantages, yet few reports have compared the efficacy of these approaches in the treatment of cervical spinal cord injury. Thus, the motivation and novelty of this study is to evaluate the efficacy of the two surgical approach on cervical spinal cord injury from multiple aspects. In this study, we retrospectively analyzed the clinical data of 180 patients with cervical spinal cord injury admitted to our hospital from June 2019 to June 2021. The curative effects of anterior subtotal vertebrectomy and posterior total laminectomy were compared, and a comprehensive comparison was made from the level of multiple postoperative indicators to provide a reference for the selection of clinical treatments and surgical methods.

## 2. Materials and Methods

### 2.1. Subjects

The clinical data from 180 patients with cervical spinal cord injury admitted to our hospital from June 2019 to June 2021 were retrospectively analyzed. The patients were divided into an anterior approach group (*n* = 89) and a posterior approach group (*n* = 91) based on the treatment method. The inclusion criteria were as follows: (1) cervical spinal cord injury based on the history of trauma, clinical manifestations and signs of patients, cervical dislocation in combination with cervical X-ray and CT examination, and MRI showing changes in spinal cord signals at different segments of the cervical spinal cord; (2) no history of cervical spine or neck surgery; (3) compete clinical data; (4) cases with follow-up times longer than 6 months. The exclusion criteria were as follows: (1) patients with severe head and chest trauma combined with other fractures; (2) patients with unstable vital signs; (3) patients with other serious diseases complicated with tumors; (4) pregnant or lactating patients; (5) patients with incomplete follow-up information.

In the anterior approach group, there were 53 males and 36 females that had an average age of 41.3 ± 6.3 years and included 42 traffic accident injuries, 28 tumbling injuries, 12 fall injuries, and 7 crush injuries. In the posterior approach group, there were 54 males and 37 females that had an average age of 42.2 ± 7.6 years and included 47 traffic accident injuries, 23 tumbling injuries, 11 fall injuries, and 10 crush injuries. The general characteristics of the two groups of patients were not significantly different, as gender (*χ*^2^ = 0.0008, *P* = 0.9771), age (*t* = 0.8640, *P* = 0.3888), and classification (*χ*^2^ = 1.3223, *P* = 0.7239).

### 2.2. Therapeutic Methods

Upon admission to hospital, all patients were given a neck brace for neck immobilization with symptomatic supportive treatment using drugs such as glucocorticoid and mannitol. Patients in the anterior approach group were treated with anterior subtotal vertebral resection, decompression, and internal fixation. The patients were placed in the supine position, and the surgical incision was made on the right side of the anterior part of the neck. The skin and tissues were cut layer by layer until the front of the vertebral body was reached to fully expose the responsible segment. The surgical segments were again determined based on the intraoperative C-arm X-ray results (Philips Medical Systems Nederland B.V. Medical IT), and the decompression range was again determined based on the patient's preoperative cervical CT or cervical MRI findings. After localization, the vertebral body was resected and the corresponding injured disc was resected completely. Autologous bone was implanted with titanium mesh and installed in the decompression area. The vertebral body in the decompression area was fixed with a locking internal fixation plate. A drainage tube was placed, and the incision was sutured after confirmation.

Patients in the posterior approach group were treated with posterior total laminectomy, decompression, and internal fixation. The patients were placed in the prone position, and continuous traction was performed on the skull to maintain the neutral position of the neck. In the absence of compression of both eyes, a posterior median cervical incision was made layer by layer to fully expose the vertebral plate and lateral masses. After localization, a lateral mass screw was inserted and the connecting rod was fixed. The ligamentum flavum and all vertebral plates of the corresponding segment were resected. A drainage tube was placed, and the incision was sutured after confirmation.

Patients in both groups were returned to the ward with a neck brace after surgery and underwent routine ECG monitoring, atomization of sputum, nasogastric gastrointestinal nutrition, oxygen inhalation, and sputum aspiration. Changes in the vital signs of patients were closely monitored, and particular attention was given to spinal cord function and incision drainage. In cases in which the patient had dyspnea or asphyxia, or the muscle strength of the limbs was significantly decreased or paralyzed compared to before surgery, the incision surface dressing was opened immediately to assess swelling and cyanosis on the skin around the incision and to squeeze the incision to ensure the drainage fluid was smooth.

### 2.3. Observational Indicators

1. The perioperative indicators were compared between the two groups, including operation times, blood loss, wound lengths, the length of hospital stay, and surgical costs
2. The pain degree of patients in the two groups was compared. The visual analogue scale (VAS) [15] was used to score the degree of pain in the upper limb and neck before and after 1, 6, and 9 months of follow-up. On a scale of 0 to 10, the patients were asked to express the degree of pain according to how they felt using the following scoring criteria (0: painless; <3: mild pain, tolerable; 4-6: moderate pain, increasing but tolerable, 7-10: severe pain, increasing and unbearable)
3. Cervical spinal cord function was evaluated using the JOA [16] scoring system before surgery and at 1, 6, and 9 months of postoperative follow-up. The items included daily activity restriction, and clinical and subjective symptoms that scored a total of 17 with lower scores indicating higher levels of patient dysfunction
4. Improvements in cervical function were evaluated by NDI [17] before surgery and at 1, 6, and 9 months of follow-up and included 10 items (pain degree, living conditions, lifting, reading, headache, concentration, work, driving, sleep, and entertainment). Each item was allocated 5 points and the total score was 50 points with higher scores indicating higher levels of cervical spine dysfunction
5. The ASIA spinal nerve function score [18] was used to evaluate the motor and sensory cervical nerve function before surgery and at 1, 6, and 9 months after the surgery. The score is divided into 5 grades as shown below. Grade A (1 point): complete spinal cord injury with no motor or sensory function in the sacral segment; grade B (2 points): incomplete spinal cord injury with sensory function and without motor function; grade C (3 points): incomplete spinal cord injury with sensory and motor functions and muscle strength below grade 3; grade D (4 points): Incomplete spinal cord injury with sensory and motor function, and muscle strength above grade 3; grade E (5 points): normal sensory and motor function, normal muscle strength
6. Postoperative complications were counted including incision and lung infections, esophageal injury, hoarseness, and loosening of internal fixation
7. Before surgery and at 9 months after surgery, the SF-36 scale [19] was used to evaluate the quality of life of patients including physiological and physical function, pain, social function, energy, emotional function, and mental and overall health. The score of each dimension was 100 points with a higher score indicating a better quality of life

### 2.4. Statistical Analysis

The approximate normal distribution method in PASS 15.0 software was used to calculate the sample size. A preliminary analysis of the data was performed with a two-sided test level with an *α* value of 0.05 and an odds ratio (OR) of 2.0. Assuming that the rate of surgical complication is 22% and 80% power is required, the sample size of each group should be at least 68 cases with 136 cases in total. Based on a 20% loss rate, each group, therefore, needed to include at least 85 cases totaling 170 cases.

SPSS 25.0 software (SPSS, Inc., Chicago, USA) was used for statistical analysis. Counting data were represented as *n* (%), and a chi-square test or Fisher's precise test was used for comparison between groups. Measurement data were expressed as the mean ± SD. For measurement data, a paired sample *t*-test was used for intragroup comparison, and a repeated ANOVA was used for intergroup comparisons. Bonferroni correction was used for post hoc multiple comparisons. *P* values of <0.05 were considered statistically significant.

## 3. Results

### 3.1. Comparison of Perioperative Indicators

According to the comparisons of the perioperative indicators of the two groups, there was no significant difference in the length of hospital stay, between the two groups. In contrast, the intraoperative blood loss was higher in the posterior approach group compared to the anterior approach group. Also, the wound diameters and operation times were longer, and the operation cost was higher in the posterior group than in the anterior approach group (all *P* < 0.05, Table 1).

### 3.2. Comparison of the Degrees of Pain

**Figure 1:**
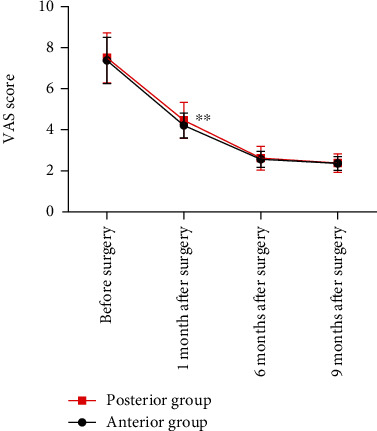
Comparison of the degree of pain between the two groups. (^∗∗^*P* < 0.01).

The pain degree in the two groups was compared using the VAS score. The results showed that the VAS scores in both groups had a decreasing trend. One month after surgery, the VAS score in the anterior approach group was significantly lower than that in the posterior approach group (*P* < 0.05). The VAS scores before surgery and at 6 and 9 months after surgery showed were not significantly different between the two groups (all *P* > 0.05, Figure 1).

### 3.3. Comparison of JOA Scores

The JOA scores of the patients in the two groups were compared before surgery and at 1, 6, and 9 months after surgery. The results showed that the JOA scores of both groups at 1, 6, and 9 months after surgery were significantly higher than before surgery (all *P* < 0.05); however, no significant difference was observed in the JOA scores between the two groups at each time point (*P* > 0.05, Table 2).

### 3.4. Comparison of NDI Scores

The NDI scores of the two groups were compared before surgery and at 1, 6, and 9 months after surgery. The results showed that the NDI scores at 1, 6, and 9 months after surgery in both groups were significantly lower than before surgery (all *P* < 0.05); however, no significant difference was observed in the NDI score between the two groups at any time point (*P* > 0.05, Table 3).

### 3.5. Comparison of ASIA Scores

The ASIA scores of the two groups of patients were compared before surgery and at 1, 6, and 9 months after surgery. No significant differences in the ASIA score were observed between the two groups of patients at each time point (*P* > 0.05, Table 4).

### 3.6. Comparison of Complications

In the anterior approach group, there were 3 cases of infected incisions, 2 cases of lung infection, 2 cases of esophageal injury, 10 cases of hoarseness, and 2 cases of internal fixation loosening, with a total incidence of 21.2%. In the posterior approach group, there were 5 cases of infected incisions, 2 cases of lung infection, 5 cases of esophageal injury, 10 cases of hoarseness, and 3 cases of internal fixation loosening, with a total incidence of 27.4%. There were no significant differences in the incidence of complications between the two groups (all *P* > 0.05, Table 5).

### 3.7. Comparison of Quality of Life

The pre- and postoperative quality of life of the patients was scored based on physiological and physical function, bodily pain, social function, energy, emotional function, and mental and general health. The results showed no significant differences in the scores of quality of life indicators between the two groups before and at 9 months after surgery (both *P* > 0.05, Table 6).

## 4. Discussion

Cervical spinal cord injury is a form of central nervous system injury that involves permanent complete or partial loss of sensory functions and is mainly caused by direct or indirect injury [20]. In the case of high-energy injuries, the spinal cord is squeezed and deformed after traumatic force resulting in a series of pathological changes [21]. Currently, the treatment of cervical spinal cord injury involves surgical decompression or drug therapy. Whilst surgery cannot change the degree of the primary spinal cord injury, it can restore the stability of the cervical spine and relieve spinal cord compression to alleviate secondary spinal cord injury. This study analyzed the differences in the curative effects of anterior cervical corpectomy and fusion and posterior total laminectomy in the treatment of cervical spinal cord injury.

Our results showed no significant differences in the length of hospital stay between the two groups, yet the amount of blood loss in the posterior approach group was larger than in the anterior approach group. Also, the wound diameters and operation times in the posterior approach group were longer than in the anterior approach group. The surgical cost was higher in the posterior approach group than in the anterior approach group. In a meta-analysis to evaluate the efficacy of anterior vertebrectomy and posterior laminoplasty in the treatment of posterior longitudinal ligament calcification, it is found that in terms of operation time and bleeding volume, meta-analysis of studies showed that the operation time of posterior 1aminoplasty was shorter than that of anterior cervical corpectomy and fusion, and there was no significant difference in intraoperative bleeding volume between the two groups [22, 23].

Decompression and internal fixation of subtotal vertebral body resection is a common anterior approach operation for cervical spinal cord injury. Anterior approach surgery has the advantages of providing immediate stability, easy exposure of the surgical site, and a large range of exposure. Herniated discs and anterior margins of fractured vertebrae can be removed under direct vision, and hanging fragments can be removed without injury to the spinal cord and nerve roots. In the anterior approach surgery, the path is mostly muscle space involving the medial space of the sternocleidomastoid muscle and the medial space of carotid sheath [24] with almost no fat and loose anterior tissue. For skilled surgeons, loose fascia and space undoubtedly reduce the time required for exposure. In addition, the anterior approach has less room for exposure and, accordingly, minimal bleeding without damaging large vessels. For the posterior approach, the muscle tissue behind the vertebral body is more developed, the structure is close, the blood supply is more abundant, and there are more small vessels [11]. Although the structure is simple, it takes more time to better expose the operation site and control the amount of blood loss.

The VAS scores in patients in the posterior approach group were significantly higher than the anterior approach group one month after surgery. These data may be because the wound diameter of the posterior approach group was larger than the anterior approach group and the range of osteotomy and grooving during surgery was often larger. Subsequently, the recovery of the cervical spine and neurological function in the two groups after surgery were compared. The JOA, NDI, and ASIA scores of both groups at 1, 6, and 9 months after surgery were higher than before surgery but the differences were not statistically significant. These results indicate that both surgical methods can quickly relieve the symptoms of spinal cord compression, promote the rapid recovery of spinal cord function, and improve the cervical spine and neurological function recovery of patients.

Patients with cervical spinal cord injury are most concerned with the recovery and improvement of limb function after treatment particularly the recovery of hand function as it is closely related to life and work. It has been reported that patients with cervical spinal cord injury can improve hand function using functional exercise equipment [25] and by changing hand activity patterns after treatment [26]. In this study, no significant differences in the complication rate and quality of life score were observed between the two groups. We also found that hoarseness occurred in a large number of cases caused mainly by edema and short-term ischemia from excessive intraoperative traction of the esophagus, trachea, and nerves. As this is a short-term injury, in most cases, the associated symptom cases will be relieved after a period of aerosol inhalation and symptomatic treatment with few permanent injuries.

Whilst our data are robust, this study had several limitations. In addition to the influence of the surgical approach on cervical spine function recovery, other physical function recoveries such as hand function were not been observed. Also, our study used a relatively small sample size. Although the observed differences are statistically significant, the long-term efficacy was not determined due to a short follow-up period. Further validation in a larger patient cohort with long-term clinical follow-up is necessary.

## 5. Conclusion

In conclusion, both anterior cervical corpectomy and fusion and posterior total laminectomy can effectively restore neurological function and achieve ideal therapeutic efficacy in the treatment of cervical spinal cord injury. However, the perioperative indicators of the former are better than that of the latter, and the surgical cost of the former is lower than that of the latter. Therefore, surgical methods should be selected according to the imaging findings, the general condition of patients, and individual economic status.

## Figures and Tables

**Table 1:** Comparison of perioperative indexes between the two groups.

	Intraoperative blood loss (mL)	Time of operation (min)	Wound diameter (mm)	Length of stay (d)	Surgery expense (CNY)
Anterior group (*n* = 89)	165.48 ± 18.93	108.18 ± 10.53	32.21 ± 2.02	9.36 ± 2.01	6908.21 ± 468.08
Posterior group (*n* = 91)	324.88 ± 44.45	115.07 ± 11.98	33.74 ± 2.84	10.00 ± 2.65	7393.50 ± 556.73
*χ* ^2^/*t*	31.1770	4.0949	4.1569	0.3511	76.7487
*P*	**<0.0001**	**0.0001**	**<0.0001**	0.0700	**<0.0001**

**Table 2:** Comparison of JOA scores between the two groups.

	Before surgery	1 month after surgery	6 months after surgery	9 months after surgery
Anterior group (*n* = 89)	8.89 ± 1.03	9.92 ± 0.75^∗^	11.35 ± 1.11^∗^	12.55 ± 1.45^∗^
Posterior group (*n* = 91)	9.05 ± 1.25	10.09 ± 0.99^∗^	11.17 ± 1.26^∗^	12.38 ± 1.41^∗^
*χ* ^2^/*t*	0.9361	1.2965	1.0162	0.7975
*P*	0.3505	0.1965	0.3109	0.4263

Note: ^∗^*P* < 0.05 vs. the same group before surgery.

**Table 3:** Comparison of NDI scores between the two groups.

	Before surgery	1 month after surgery	6 months after surgery	9 months after surgery
Anterior group (*n* = 89)	25.19 ± 2.90	19.23 ± 2.26^∗^	15.35 ± 2.14^∗^	12.62 ± 3.29^∗^
Posterior group (*n* = 91)	24.96 ± 3.12	18.67 ± 2.64^∗^	15.76 ± 2.25^∗^	13.23 ± 3.89^∗^
*χ* ^2^/*t*	0.5120	1.5273	1.2522	1.1348
*P*	0.6093	0.1285	0.2121	0.2580

Note: ^∗^*P* < 0.05 vs. the same group before surgery.

**Table 4:** The ASIA score.

	Before surgery	1 month after surgery	6 months after surgery	9 months after surgery
Anterior group (*n* = 89)	2.45 ± 0.24	3.16 ± 0.31^∗^	3.96 ± 0.25^∗^	4.33 ± 0.44^∗^
Posterior group (*n* = 91)	2.51 ± 0.31	3.21 ± 0.32^∗^	4.03 ± 0.29^∗^	4.40 ± 0.44^∗^
*t*	1.4498	1.0819	1.7329	1.0672
*P*	0.1489	0.2808	0.0848	0.2873

Note: ^∗^*P* < 0.05 vs. the same group before surgery.

**Table 5:** Comparison of the incidence of complications between the two groups.

	Infected incisions	Lung infection	Esophageal injury	Hoarseness	Internal fixation loosening	Total incidence rate
Anterior group (*n* = 89)	3 (3.4)	2 (2.2)	2 (2.2)	10 (11.2)	2 (2.2)	19 (21.2)
Posterior group (*n* = 91)	5 (5.5)	2 (2.2)	5 (5.5)	10 (10.9)	3 (3.3)	25 (27.4)
*χ* ^2^/*t*						0.9137
*P*						0.3391

**Table 6:** Comparison of quality of life scores between the two groups.

	Physiological function		Physical function		Bodily pain		Social function	
	Before surgery	9 months after surgery	Before surgery	9 months after surgery	Before surgery	9 months after surgery	Before surgery	9 months after surgery
Anterior group (*n* = 89)	64.51 ± 6.29	81.33 ± 5.81^∗^	61.12 ± 6.61	81.26 ± 6.52^∗^	60.63 ± 5.30	80.49 ± 5.05^∗^	64.81 ± 7.04	84.54 ± 5.95^∗^
Posterior group (*n* = 91)	64.89 ± 7.29	82.82 ± 6.15^∗^	62.24 ± 5.38	81.70 ± 7.85^∗^	61.14 ± 6.35	80.53 ± 4.20^∗^	64.03 ± 6.72	84.19 ± 5.31^∗^
*χ* ^2^/*t*	0.3741	1.6701	1.3480	0.4086	0.5843	0.0578	0.7605	0.4166
*P*	0.7088	0.9667	0.2136	0.6833	0.5597	0.9539	0.4479	0.6775

	Energy		Emotional function		Mental health		General health	
	Before surgery	9 months after surgery	Before surgery	9 months after surgery	Before surgery	9 months after surgery	Before surgery	9 months after surgery
Anterior group (*n* = 89)	66.83 ± 6.40	83.56 ± 3.79^∗^	69.42 ± 6.17	84.49 ± 6.96^∗^	68.75 ± 4.33	85.26 ± 5.94^∗^	64.20 ± 5.21	85.02 ± 4.70^∗^
Posterior group (*n* = 91)	66.98 ± 6.40	83.82 ± 4.79^∗^	68.35 ± 5.15	84.12 ± 5.83^∗^	68.52 ± 5.12	85.40 ± 5.41^∗^	63.87 ± 6.70	85.26 ± 4.24^∗^
*χ* ^2^/*t*	0.1572	0.4033	1.2642	0.3870	0.3251	0.1654	0.3683	0.3599
*P*	0.8753	0.6872	0.2078	0.6992	0.7455	0.8688	0.7131	0.7194

Note: ^∗^*P* < 0.05 vs. the same group before surgery.

## Data Availability

The data used for this study have been included in the manuscript.
